# Slowing down decay: biological clocks in personalized medicine

**DOI:** 10.3389/fsoc.2023.1111071

**Published:** 2023-04-17

**Authors:** Clémence Pinel, Sara Green, Mette N. Svendsen

**Affiliations:** ^1^Centre for Medical Science and Technology Studies, Department of Public Health, University of Copenhagen, Copenhagen, Denmark; ^2^Section for History of Philosophy of Science, Department of Science Education, University of Copenhagen, Copenhagen, Denmark

**Keywords:** biological clocks, aging, decay, maintenance, personalized medicine, unknowing

## Abstract

This article discusses so-called biological clocks. These technologies, based on aging biomarkers, trace and measure molecular changes in order to monitor individuals' “true” biological age against their chronological age. Drawing on the concept of decay, and building on ethnographic fieldwork in an academic laboratory and a commercial firm, we analyze the implications of the development and commercialization of biological clocks that can identify when decay is “out of tempo.” We show how the building of biological clocks rests on particular forms of knowing decay: In the academic laboratory, researchers focus on endo-processes of decay that are internal to the person, but when the technology moves to the market, the focus shifts as staff bracket decay as exo-processes, which are seen as resulting from a person's lifestyle. As the technology of biological clocks travels from the laboratory to the market of online testing of the consumer's biological age, we observe shifting visions of aging: from an inevitable trajectory of decline to a malleable and plastic one. While decay is an inevitable trajectory starting at birth and ending with death, the commercialization of biological clocks points to ways of stretching time between birth and death as individuals “optimize” their biological age through lifestyle changes. Regardless of admitted uncertainties about what is measured and the connection between maintenance and future health outcomes, the aging person is made responsible for their decaying body and for enacting maintenance to slow down decay. We show how the biological clock's way of “knowing” decay turns aging and its maintenance into a life-long concern and highlight the normative implications of framing decay as malleable and in need of intervention.

## Introduction

This article is concerned with technologies aimed at measuring and quantifying aging, which are commonly referred to as “biological clocks.”^1^ As we unpack what the building and commercialization of such a technology entail, we examine the associated implications for how we understand aging processes and the aging person. While for a long time, aging was commonly viewed as an inescapable trajectory of decline, today's biomedicine construes aging processes as modifiable and amenable to interventions (Moreira, 2015; Blasimme, 2021). Biomedicine, and aging research more specifically, place their gaze on the molecular, by studying biological processes underpinning aging and considering how such processes can be manipulated to slow down aging (Moreira and Palladino, 2009). The notion of “healthy aging”, which is ever present in health policy recommendations about old age (World Health Organization, 2015), suggests that every person differs in their aging processes and that personalized strategies are needed to control or “optimize” aging (Lassen and Moreira, 2014). Crucially, such strategies rely on measurements of aging that can assess a person's health and his or her rate of deterioration compared to a reference population (Green and Hillersdal, 2021). Drawing on various methods, such as molecular biomarkers, measures of body composition, or questionnaires collecting anthropometric and health behavior data, these technologies promise personalized assessments of a person's biological, functional or “real age” (Moreira et al., 2020). The biological clocks discussed in this article are one example of such measurement technologies.

In our empirical field, biological clocks are technologies aiming to calculate a person's biological age, which is traditionally understood as a measure of the effect of time on a body, in contrast to a person's chronological age, which refers to how many years a person has lived (Nathan, 2021). In practice, biological clocks are based on biomarkers of aging that trace and measure molecular changes. Or as geneticist Macdonald-Dunlop et al. (2022, p. 623) put it:

We all become acquainted with visible changes that accompany aging, such as graying hair, baldness, loss of skin elasticity and worsening of posture, and that these vary noticeably amongst individuals of the same chronological age. However, there are also molecular hallmarks of aging such as telomere shortening, genomic instability and cellular senescence that also show variation in individuals of the same chronological age.

In other words, Macdonald-Dunlop et al. (2022) suggest that aging leaves molecular traces, which technologies of biological clocks aim to make visible. Biological clocks are studied for the purposes of better understanding aging at multiple biological levels, but they are also intended to help better prevent age-related diseases. In such cases, aging is understood as a risk factor for multiple diseases, and measurements of biological aging are considered possible predictors of, and intervention sites for, the speed of decay (Green and Hillersdal, 2021). Or as science scholars Müller and Samaras (2018) put it, like much of contemporary aging research, biological clocks focus on the aging body to map out and intervene upon aging processes, rather than the aged or old body.

One of the most well-known biological clocks is the epigenetic clock developed by bio-mathematician Steve Horvath and colleagues (Horvath, 2013; Horvath et al., 2014). Drawing on earlier insights that aging is related to changes in DNA methylation in cells, they used epigenomics data to precisely track CpG methylation patterns^2^ across the genome to estimate a person's biological age. Biological clocks have since been constructed using a variety of omics technologies, such as metabolomics, proteomics, microbiomics, and glycomics. The development of biological clocks is thus deeply intertwined with and dependent on the ever expanding technologies and data, which can be utilized to build a new biological clock. Common to these efforts is the understanding that molecular changes can provide information on dynamic phenomena ranging from trauma, environmental exposures, lifestyle, or socioeconomic status. By incorporating information about the nature of a person's life through factors such as lifestyle or exposures, biological clocks building on these omics technologies promise dynamic and personalized measures of our health and bodies, while leaving open the possibility of change over time (Knoppers et al., 2021). However, one should not just take for granted the realization of such promises. Biological clocks are still uncertain and controversial epistemic objects, with many actors in the field showing skepticism about their accuracy, validity, reliability, and even their purpose (Moreira, 2015).

As technologies offering personalized measures of aging based on individuals' molecular markers, biological clocks serve the broad agenda of personalized medicine—a term referring to a shift within healthcare and the medical sciences from a one-size-fits-all approach toward the tailoring of diagnostics and therapeutics to individual characteristics (Collins, 2010). For Moreira (2015, p. 20), growing interest in biological age in fact represents a shift in the ways biomedicine approaches and segments the life course, from relying only on chronological age toward “destandardized, individualized life course trajectories.” As we study biological clocks, we thus point to how technoscientific practices enable new ways of individualizing life course trajectories, which, more broadly, helps map out what personalized medicine looks like in practice.

It is also worth noting that biological clocks, while representing an expanding academic research field, also constitute a growing industry (Dupras et al., 2020). Biological clocks make commercial products, increasingly available as online services, where individuals can get tested to be informed about their “true” age through personalized age measurements. We empirically study biological clocks both in academic research and in the commercial world, drawing on ethnographic fieldwork in a research laboratory and in a commercial firm. Across these two sites, we take a specific interest in the development of such technologies. While biological clocks are becoming popular, little is known about how biological clocks are developed and commercialized as direct-to-consumer products on the online market. This means that little is known about what drives the technologies available today, what resources and methods are utilized to create biological clocks, or what discourses and concepts are mobilized to enable possibilities and address specific concerns. In this article, we focus on the imaginations and conceptualizations of aging that support the development of biological clocks in both the laboratory and the commercial world. Our aim is to unpack how aging processes are conceptualized and the aging person enacted in contemporary health technologies, and to discuss the implications of such technologies for understandings of individual agency over the aging trajectory. To help us do this, we draw upon the concept of decay. In the next section, we explain our use of the concept of decay to theorize the making of biological clocks.

## Decay as analytical framework

To “decay” means to decompose and deteriorate. Anything and everyone decays: bridges (Gupta, 2021), prisons (Kohn, 2021), communities (Schubert, 2021), as well as bodies and minds (Gjødsbøl et al., 2017). Decay thus constitutes a normal process that comes with being, but also one that can process at an unwanted pace. In the case of human bodily decay, anthropologist (Hage, 2021, p. 3) reminds us that one can distinguish between normal and pathological decay, whereby pathological decay is:

The decay that marks us experientially such that we end up noticing it, is a decay that is happening at what we consider an unusual rhythm, often too quickly, but sometimes too slowly, and a decay that is progressing outside the confines of where we expect it to exist.

Decay is thus defined by a norm. Importantly, norms of decay do not just “exist” as universal standards. Rather, conceptions of aging are socially constructed through particular cultures, groups, and contexts, which are themselves dependent on socially constructed standards of living. This is illustrated in the “bio-medicalization of aging” (Estes and Binney, 1989), which is tied to societal challenges of aging populations in Western societies. Moreira (2015, p. 27) exposes how the organization of biomedical research and “institutional configurations of expertise” within biomedicine in the Global North led to the now dominant understanding of aging as a medical problem to be addressed at the individual level and through biomedical interventions. In other words, norms of decay are tied to particular places, cultural contexts, and politics, which dictate at what tempo decay is deemed acceptable, as well as what constitutes appropriate ways of intervening upon it. Thus, decay has a temporality—it operates according to a pace—as well as a spatiality—it occupies and evolves in particular places. In this paper, we draw on decay as an analytical category to problematize such norms about tempo associated with the development and marketing of biological clocks, and reflect on how these norms are tied to particular places.

Although decaying buildings or communities may seem very far from decaying bodies, the conceptual framework emerging from the decay literature is helpful in excavating how bodily decay is approached in personalized medicine. From this literature, we learn that there are different processes that can lead to decay. Anthropologists (Klein, 2021; Klem, 2021) studying narratives of decay in particular social groups such as settler colonial Australia or post-war Sri Lanka distinguish between *endo-decay*, to refer to when things are thought to decompose from the inside, from *exo-decay*, whereby disintegration is understood to be caused by external factors. This distinction is hardly ever neat, and the two processes often entangle and shift from one to another. Crucially, the distinction between what counts as endo- vs. exo-decay is closely tied to norms and values about decay. In his study of urban decay in Rome, Herzfeld (2021) points out how different narratives and interpretations are mobilized to discuss the cause of urban decay, pointing to endo- vs. exo-processes. He specifically points to an ambiguity: on the one hand, citizens of Rome cherish the genteel patina of urban infrastructures in the city, investing in and restoring old neighborhoods; on the other hand, citizens are anxious about the perceived degradation of cultural and moral standards, what Herzfeld refers to as “social decay.” Interestingly, a particular interpretation of the causes of these two forms of decay is put forward: the former is deemed to be the inevitable result of time and endo processes, while the latter is attributed to increased human circulation and exposure from migrants coming into the city, and thus the result of exo-processes. In this example, the distinction between endo- and exo-decay differentiates between processes that are considered generic or determined (endo-decay) and processes that are thought to be related to human agency (exo-decay). When it comes to human bodily decay, some aspects of decay may similarly be considered endo-processes (e.g., a genetic disposition for disease) while other aspects may be considered exo-processes (e.g., diet or exercise). More broadly, this literature helps us reflect on what factors are understood to be causing decay, and how such factors are approached, studied, and intervened upon to act upon decay. In other words, it points to particular ways of “knowing” decay.

This point connects with discussions about maintenance, which is essential to stave off or slow down decay. The notion of maintenance is by some social science scholars approached as a care practice that takes into account decay and vulnerability (Tronto, 1994; De Laet and Mol, 2000).^3^ Similarly, we see maintenance as starting from the fragility of life and things and involving practical tinkering. But noting that decay not always leads to maintenance work, we explore what maintenance to resist decay entails, where and when it takes place as well as who is involved. Decay is only deemed problematic at certain times, places, and when it touches certain people, such as valued members of society. This comes to the fore in Kohn (2021) study of processes of decay in the US prison system. Prisoners may be left to decay physically, mentally, and socially in buildings that are themselves physically molding and crumbling, without many noticing or paying attention. But this would not be the case for high schoolers, especially in a well-off district—one could imagine how a rotting and molding high school building would cause outrage and be the subject of interventions to repair the visible decay. This example illustrates how maintenance to slow down decay, whether that is of buildings or of bodies, targets specific places and people, especially the Global North and the already well-off. This is something we too observed with the development of biological clocks, which are predominantly aimed at wealthy customers of the Global North (see also Müller and Samaras, 2018). More broadly, decay implies a politics, whereby some things and people are left to rot, their decay being considered normal, while others are attended to and maintained so as to stop or slow down their decay, which is deemed pathological. In addition, there are different ways of maintaining entities (Denis and Pontille, 2017). If we take the example of a high school building prone to mold, maintenance can take the shape of an organized monthly clean-up where staff and students take turns wiping out mold. But maintenance could also mean investment from regional authorities to carry out structural works on the building, with better ventilation and insulation, as an attempt to solve a more general underlying problem. As such, different approaches to maintenance put different emphases on who is responsible and who should do the maintenance work. Investigating what form of maintenance is encouraged in connection to aging is particularly telling of what is understood as factors of cause and control. As we highlight in this paper, promoting control of decay as a practice of *self-*care frames individuals as agents of responsibility for their trajectory of aging.

Finally, the concept of decay brings to our attention a particular conception of time and of the person. Bodily decay positions individuals on a trajectory of functional decline with an inevitable ending, that of death. This trajectory is marked by feelings of loss. That is, as people decay, they lose capabilities or prosperity. However, with maintenance work, a person can suspend or slow down decay in the present, which offers the promise of a longer and healthier future. The human body, while always decaying and moving toward death, is improvable and “plastic,” offering the possibility of slowing down the pace of decay. In other words, while people cannot stop their own decay from happening, it can be slowed down. It is an inevitable fate, yet one that can be monitored, managed and controlled.

In our study of biological clocks, we employ these insights to better understand how the bodily decay measured by the clocks is approached and made sense of as a problematic phenomenon that needs addressing, and crucially, to examine who and what is held responsible for decay and for enacting maintenance work to keep it at bay. As an analytical lens, the concept of decay is particularly useful because it renders visible how processes of decay (and maintenance) are inherently cultural and political, as they rest on particular understandings of what and who is valuable, and who is responsible for taking action. Drawing on the concept of decay, we thus ask: How are biological clocks developed and commercialized to assess when decay is out of tempo? What are the temporalities enacted through biological clocks and what do they mean for understandings of the person and the person's agency? Who is implicitly held responsible for managing bodily decay?

## The study

This article is based on a study investigating the contemporary data ecosystem of personalized medicine, specifically interrogating the flows (of data, people, funds, etc.) making and maintaining this ecosystem. To this end, the first author conducted ethnographic fieldwork in empirical sites connected by data, the first of which is the Wilson Lab. Based in Scotland, the Wilson Lab is an academic research laboratory well known for its database of phenotypic and genotypic data originating from a cohort of healthy volunteers from the Northern Isles of Scotland. For Jim, the PI of the lab (and many others in the scientific community), the people of the islands represent “isolated populations,” which make excellent study samples for various genetic investigations, ranging from the identification of genes involved in rare diseases to the characterization of interactions between genes and the environment (Kristiansson et al., 2008). The Wilson Lab is composed on the one hand of staff managing the cohort and curating the resulting database, and on the other hand, researchers utilizing the available data for the production of knowledge. Based on their cohort data, staff at the Wilson Lab study population and disease genetics with a focus on the genetic architecture of complex traits. While ethnographic fieldwork looked to explore the making of data in the cohort and its uses in biomedical research by the lab, the first author also came to learn more about the work of one particular team member, who was building biological clocks in an attempt to learn about aging processes.

The second site is a biotechnology company called Genos. Based in Croatia, Genos is specialized in the study of glycans—sugar molecules surrounding proteins in the blood that influence the immune system. Having developed a method for the high-throughput analysis of glycans, Genos sells analysis services to institutions conducting cohort studies for research, turning biological materials into glycomics data. As part of an EU-funded consortium, the Wilson Lab and Genos collaborated, which entailed Genos analyzing samples from the Wilson Lab, turning them into high-throughput data to be added to the Wilson Lab's database. The first author conducted ethnographic fieldwork at Genos to learn more about their work producing high-throughput omics data for a wide range of customers around the globe. During fieldwork, the first author learned about a biological age test, called GlycanAge, which they sell online to individual customers for a fee. The test was developed by Genos staff at the bench, based on their expertise in the analysis of glycans, but its running and marketing are now overseen by a dedicated team of three employees, led by a project manager.

The first author conducted ethnographic fieldwork in October 2019 at Genos and February 2020 at the Wilson Lab. In both sites, it involved observing data practices. The first author observed laboratory staff working at the bench with biological materials, witnessed database workers processing omics data, or watched researchers analyze large datasets using computational methods. Fieldwork also entailed sitting in meetings, attending seminars, and sharing lunch or coffee breaks with team members. The first author gained consent from participants by cultivating ethical mindfulness (Sleeboom-Faulkner et al., 2017), actively situating ethics within the research process, and securing relationships of trust with participants. The ethnographic data consists of field notes from participant observation, informal conversations with staff, reflections from meetings, as well as in-depth semi-structured interviews with members of the teams carried out by the first author (seven at Genos and 12 at the Wilson Lab). All informants were pseudonymized. We then coded all materials thoroughly following network thematic analysis (Attride-Stirling, 2001). We specifically draw upon interview data with the people in the two laboratories who were involved in building and commercializing biological clocks. In addition, we draw upon promotional materials from the GlycanAge website.

In what follows, we discuss our empirical results. We start in the laboratory to unpack how researchers develop their biological clocks by drawing out the norm of decay against which individuals are assessed, analyzing the particular conception of aging—as an inescapable and uncontrollable trajectory—that is enacted through this work. Then, we follow how biological clocks are transformed into wellness technologies that can be sold on the market, and how this transformation shifts understandings of aging. Finally, we discuss the particular forms of maintenance that are advocated to control the forces of decay identified through the biological clocks, pointing to how it turns aging into a space of intervention, while it transforms the individual into the agent of cause and control.

## Aging as endo-decay

The development of a biological clock starts with the making of what practitioners call a “baseline,” that is, a basic standard that can serve as a comparison or control. A baseline is needed to draw out the norm of decay against which individuals can be assessed and their biological age calculated. This norm suggests both the appropriate effect of time on human bodies at specific time points and a pace at which decay happens over time. In this section, we discuss what goes into the building of a norm for biological decay and analyze what this norm tells us about understandings of aging processes and the aging person.

Establishing the norm for biological age first means mobilizing population data. At the Wilson Lab, PhD student Anna drew on the cohort data the lab owns and manages so as to develop a number of biological clocks. The population recruited in the cohort is a general population, all of whom share roots in the Northern Isles of Scotland. They are predominantly healthy individuals. To study this population, Anna used particular sets of data, from nine different omics technologies, including epigenomics, glycomics, proteomics, and metabolomics. She explains:

Particularly with [cohort name], not all two thousand, but over half, a thousand people have all of these multiple omics assays, like protein and certain lipids, and that sort of thing. And we want to have a measure of biological age, measure how your body deteriorate differently from just how old you are. People mainly looked at DNA methylation data, but a couple of people have looked at other types. And because we are in the position of having different data than other people [have], we want to see if you can compare whether actually you could, so that's my project. (Anna)

Anna drew on the availability of data at the Wilson Lab—high numbers of individuals and data of different sorts—to build a baseline for biological age. Crucially, Anna focused her attention on some data rather than others: she explored various omics data to trace the effect of time on human bodies, while leaving out other data also available, such as individuals' clinical history. The choice to focus on omics data alone was driven, first, by the epistemic approach of the lab when it came to the study of aging, which was to learn about aging processes by tracing molecular markers. This reflects current scientific strategies for monitoring and measuring aging based on molecular signs known as “aging biomarkers” (Green and Hillersdal, 2021). Second, the focus on omics data alone can be explained by the availability of different omics data in the lab. As mentioned, the Wilson Lab invested heavily in the development of a rich database composed of phenotypic and omics data from the people living in the Northern Isles of Scotland. For analysts like Anna, these data can be mobilized as a resource to study different molecular mechanisms, such as telomere shortening, genomic instability and cellular senescence, as well as for identifying molecular markers of biological decay that can be used as biological clocks to measure aging.

Also noteworthy is how omics data are approached and interpreted by Anna. Omics technologies are widely considered to be capable of capturing, at a molecular level, the impact of environmental exposures and individuals' experiences such as trauma or socioeconomic status (McGuinness et al., 2012; Pinel et al., 2018; Knoppers et al., 2021). This potential was indeed recognized by Anna and her peers. However, what surprised us was that in practice, when developing biological clocks, the role of environmental factors shaping their omics data was not explicitly studied. While the researchers acknowledge that environmental factors influence processes of aging, their analysis of omics data does not present or explore environmental factors as difference-makers. Rather, omics data are studied for what they can tell them about the internal molecular processes leading to a person aging. In this process, the measurement of biological age becomes disconnected from the environment. This observation echoes critical social science analyses of epigenetics (e.g., Lock, 2013; Chiapperino, 2021). Authors discuss how scientists navigate this post-genomic science, where biology is assumed to be open to the environment and plastic, by pointing to new forms of reductionism. They specifically show that while research in epigenetics pays attention to the social and material environment by producing an “embedded body” (Niewöhner, 2011: 291) imprinted by its environment and its past, in practice aspects of the social world are bracketed and situated in “quasi-natural experimental system”. In the case of the Wilson Lab, the norm of biological age constructed turns aging into an internal process. With their focus on the molecular mechanisms of aging traceable through sophisticated omics technologies, researchers conceptualize aging as processes of endo-decay.

Similarly to the Wilson Lab, at Genos, the available data drive the building of the baseline. The baseline for their biological age test is derived from their work analyzing samples and producing glycan data on behalf of cohorts and research institutes around the world, who approach glycans as causal factors for many diseases (e.g., rheumatoid arthritis, lupus, etc.). Katja, the GlycanAge product manager, who oversees the development and marketing of their biological clock, explains in an interview: “This is how we use older research; GlycanAge is like a commercial product based on all the research that has been produced at Genos so far.” Over the years, through a series of collaborative arrangements and contracts, Genos analyzed samples from about 100,000 individuals. Genos researchers draw on the resulting glycomics data to build a norm of biological age. According to the GlycanAge website, they understand aging as “the accumulation of damage in your body over time, caused by a long term over-activation of the immune system.” In that sense, they too studied aging at a molecular level, by tracing molecular changes linked to glycans.

Using the biological clock to measure an individual's biological age requires that multiple baselines are developed, as there is no universal molecular measurement of biological aging time. In addition to age, Genos staff also differentiate their data populations according to sex and ethnicity. The ethnic origins of their population data were often mentioned by Genos staff, and it constituted an important biological parameter when building the norm of biological age. In part, this is because Genos provides analysis services for cohorts around the world, and as a result, the population they curate through their data has varied origins. However, as we learn from Katja in the quotation below, the building of several baselines according to ethnicity was not only biologically justified, but also commercially motivated:

The idea is that since glycans are in a way dependent on the genes, different ethnicities have different IgG glycosylation patterns. So ideally, whenever we move to a new market we would need to define a new baseline. … Sometimes we try to cope: if the changes are not big, then we try to correct that on the data analysis level, but sometimes you cannot do that. As we spread worldwide we would definitely need… This is something we always include in the business development plan. If we go for China we first need to make a baseline for China. … Whenever we change markets or adapt to new markets, we need to develop new baselines. Like, you can use the same baseline when you're comparing Austrian and Croatian population, or like you know, something that is 300 kilometers apart, but you cannot move to Mozambique with a Croatian baseline. (Katja)

To be able to deliver testing services to individuals in all parts of the world with different ethnic backgrounds, Genos needs several baselines. Concerns about consumer markets drive the building of baselines and their efforts at including diversity. We find Genos's approach to data collection and the building of baselines to contrast with contemporary discussions about the health “data gaps,” whereby the data collected in health data infrastructure do not capture certain portions of the population. In such discussions, efforts to address the health “data gap” emphasize inclusion and justice as the motivation (Shim et al., 2022). At Genos, their approach to addressing “data gaps” seems to be directly driven by the identification of commercial market gaps.

In practice, having several baselines according to ethnicity is used to compare individuals to the “right” biological age trajectory. Individuals are plotted against a baseline according to their own ethnicity, as well as their sex. Or as Katja further puts it in an interview: “What is necessary for the test is actually the gender and the ethnicity, so we can choose which baseline we plot you on.” “Plotting individuals on the baseline” means comparing individuals' glycan profile to the norm. Katja continues:

Then according to your IgG glycan, or glycosylation pattern, we estimate for, you know, females this type of glycosylation pattern corresponds to a Caucasian female of 53 years old, for example. And this is what you get as a read-out. (Katja).

The number Katja mentions here is a person's biological age as it reflects the molecular pattern of the person compared to the average population. However, that number only makes sense when compared to the person's chronological age—that is, by comparing how “old” your body is to how many years you have actually lived. By comparing the two numbers, analysts come to differentiate normal from pathological decay, whereby any biological age above a person's chronological age is deemed pathological—it signifies decay that is out of tempo. When analyzing samples, researchers place individuals on the trajectory of aging, and their position below or above the trajectory decides on their individual pace of decay. This resonates with findings from Moreira et al. (2020), who show that individual users of biological clocks, instead of substituting chronological age for biological age, estimate the difference between the two in order to qualify their life trajectory. In our case, we see how measures of biological age coexist with, and are even dependent on, measures of chronological age. It is by comparing the two measures that one can determine when decay is out of tempo and offer personalized assessments of possible interventions, as we will show below.

We want to draw out a few lessons about the development of biological clocks in the cases presented. First, what unites the laboratories is the great extent to which the available data determine how they develop their biological age clock and baseline. The design of the model for biological age does not come before data. Rather, data availability drives them to decide what biological clock to design and develop. In this sense, their work building baselines based on data availability resonates with the concept of “data-driven” research, which captures research thought to be led by the generation and collection of vast quantities of data in order to identify new processes and phenomena. However, while developing hypotheses from patterns identified in large datasets may be data-driven, decisions still have to be made about what data to include, how to classify data, and how to interpret data and resulting models (Kell and Oliver, 2004; Leonelli, 2012). Similarly, biological clocks are developed from decisions on the most salient features of data populations, as well as scientific and normative norms for the interpretation and use of biological clocks.

This takes us to our second point, which is that baselines for biological age are constructed. They are the result of labor, resources, and choices. This resonates with critical discussions of the reference problem, which point to the implications of pragmatic choices concerning the selection of reference populations (Green and Hillersdal, 2021; Binney, 2022). In our case, the baselines are shaped by several factors, including data availability, a particular epistemic strategy to study aging, as well as considerations about the intended consumer populations and markets. Depending on what goes into the baseline, radically different biological clocks leading to various results can be produced. This point confirms the epistemic uncertainty about what is measured by biological clocks, which we hinted at in the introduction.

Third, across the two laboratories, their respective attempts at building baselines for biological age draw out a particular vision of aging. Namely, aging is seen as the result of endo-processes of decay, which can be studied molecularly using sophisticated omics technologies. Aging follows a unidirectional and inevitable trajectory, which begins at birth and ends with death, thus echoing the historically common view of aging as an inescapable trajectory of decline (Grmek, 1957; Gjødsbøl and Svendsen, 2019; Blasimme, 2021). In this vision, aging is a measurable process tied to molecular markers at specific time points, which means that individuals can be placed along that trajectory and their own aging measured. While aging is understood as a normal and physiological process, at times aging can become pathological. In the vision of aging enacted by Wilson Lab and Genos staff, what we also find striking is what becomes invisible—namely the role of socio-economic factors shaping longevity and life expectancy. While aging is situated at the molecular level and tied to internal and biological processes, the “social” roots of aging, with factors such as living or working conditions, are bracketed. As practitioners place the emphasis on the molecular and bracket the social, they enact a very particular way of knowing decay. Crucially, they conceptualize decay as an internal process tied to biological patterns and differences, while the person decaying is depicted as isolated from the immediate and wider environment. In other words, scientists turn aging into a de-socialized and de-contextualized phenomenon of the inner body.

## Aging as exo-decay

Biological clocks, while born in the laboratory in an epistemic attempt to better understand the molecular underpinnings of aging and biological decay, travel beyond the laboratory as products that can be sold to individual customers and wellness businesses. In this section, we discuss how biological clocks are packaged as direct-to-consumer tests, and show how the making of a biological clock into a product shifts understandings of aging from an inescapable trajectory, to a malleable and controllable one over which individuals have responsibility and agency.

At Genos, the biological clock designed in the lab was turned into a patented product called GlycanAge. The test is marketed as a general health and wellness test, rather than being aimed at any particular disease. Or as their website states, taking the GlycanAge test means “start[ing] your wellness journey.” Consumers are also informed that:

GlycanAge is your key to healthy aging. It is the only biological age test that accurately measures your unique response to lifestyle change. (GlycanAge website, November 2022)

Yet, the researchers behind GlycanAge caution against interpreting the biological clock as a diagnostic test. As Katja, the GlycanAge product manager, explains:

So it is basically a test that is intended not to diagnose anything. Because it is too unspecific because it basically measures the glycans on IgG that are related in some kind of chronic low-grade inflammation, mostly. That is associated with aging and aging is related to diseases. And therefore basically what you measure, these glycans on this IgG molecule, it's one of the most common analyses we actually do here, it's like bread and butter. We try to assess what is this inflammation level in your body and which age group it actually corresponds to. (Katja)

The differences between the two quotations highlight how the accuracy of measurement is not necessarily matched by conceptual precision of what is being measured. We grasp from Katja the particular pathway through which glycans are approached as proxies for inflammation, which is itself a proxy for aging, which again constitutes a risk factor for multiple diseases. In other words, for Genos researchers constructing their biological clock, glycans are connected, in a long chain of relationships, to various age-related diseases or disease risk. This comes up in Genos's research publications, many of which are concerned with particular diseases, such as COVID-19, cardiovascular diseases or cancers, and how glycans can be understood as causal factors impacting the individual's susceptibility to, or response to, such diseases. However, when glycans are studied in order to develop the GlycanAge biological clock, the relationship between glycans and specific diseases is broken up: glycans are framed as potential predictors for general processes of inflammation and aging, while researchers are hesitant to reach conclusions about causality. This approach resonates with how Anna, at the Wilson Lab, describes her work developing biological clocks:

I'm attempting to treat them as potential biomarkers, and not check for causality and because I'm trying to, eh, trying to treat them all as sort of as naïve predictors, with not really any hypothesis about which ones should help predicting age. What I have done is I have just standardized all the measures, so they are all, they will all be on the same scale, in the hope that it then cancels out the fact that they were all in different units. … Because, again, it's just predicting and not looking for causality. It's a lot of association that are just associations, they are indicative, they are not really proving anything. (Anna)

Biological clocks are here treated as “naïve predictors” of age rather than technologies that can tease out causal patterns between aging and diseases. Staff look to learn how individuals' bodily decay compares to the average population, and come up with a number to reflect that pace. If individuals' decay is deemed pathological, researchers are not looking for causality in diseases. In part, that's because staff find that interpreting the number they produce is difficult. This is something Anna mentions in an interview:

At the moment we find that tackling that number is quite easy, but whether that number is meaningful in prognosis is probably more difficult. That's what I have been doing and it's a lot of “meh” [i.e., inconclusive] results, which is not great. [Laughs] … The aim would to be able to say this number, say plus 5—if you're predicted to have the health of an average 55-year-old, if you're only 50, … that number plus five would indicate that you're more likely to have a cardiac event or develop this disease. But at this moment, it is not also always showing that. And the effect sizes are tiny. So, actually, what we found is that, very normal things like the normal blood test you would have at your GP is more indicative of that, than very fancy assays. (Anna)

While Anna's PhD project is entirely dedicated to the development of biological clocks, she shows ambivalence about the technology. Specifically, she suggests here that there is uncertainty in the results produced by the biological clocks. Because of such uncertainty, researchers prefer to approach the biological clock as merely revealing associations with aging. But we also learn that, for Genos, focusing on aging, rather than diseases, is strategic. As Katja explains, they were able to formally disconnect GlycanAge from diseases by avoiding a clinical label:

We deliberately opted out of clinically validating this as a clinical test. We even went so far as to ask for the Croatian counterpart of FDA to issue us a statement that we are not a clinical diagnostic test. (Katja)

Making sure GlycanAge is not labeled as a clinical test is important because it keeps Genos out of the more tightly regulated space of pharmaceuticals and medical devices (Simon et al., 2022). As a health test rather than a diagnostic test, GlycanAge is made to fit into the ever-expanding wellness and lifestyle market. While pathology is ever present in Genos's work, and in the references on their webpage to multiple scientific publications in biomedical journals, their commercial strategy to focus on wellness exemplifies the very thin line that exists between diagnostic tools and wellness technologies.

This shifting and often fluid relationship between biological clocks and diseases also comes across when considering the type of individual Genos targets and studies. On the one hand, in the laboratory, when Genos researchers analyze samples and turn them into glycomics data, they mostly study sick individuals who are part of disease-specific cohorts. On the other hand, when they develop and market their GlycanAge test, the individuals they focus on are healthy customers. Specifically, they have a particular healthy individual in mind. From Katja, we learn that this individual is first a customer, who is “mostly over 40” and “in the upper spending bracket, because the test is now 350 euros.” Then, from a conversation with Ivan, the CEO of Genos, we learn that this individual is aware of his or her own health “risks” and how “changing their lifestyle” can reduce those risks. We see here how norms of decay are related to what Chiapperino and Tengland (2015) term a “new wave of empowerment”, which promote proactive civic agency and private voluntarism in health promotion. In fact, in their promotional material, the GlycanAge test is presented as a tool for self-empowerment and improvement:

Discovering your biological age will provide you with the confidence that your current lifestyle is optimal, or empower you to make changes if there could be room for improvement. (GlycanAge website, November 2022)

**Figure 1:**
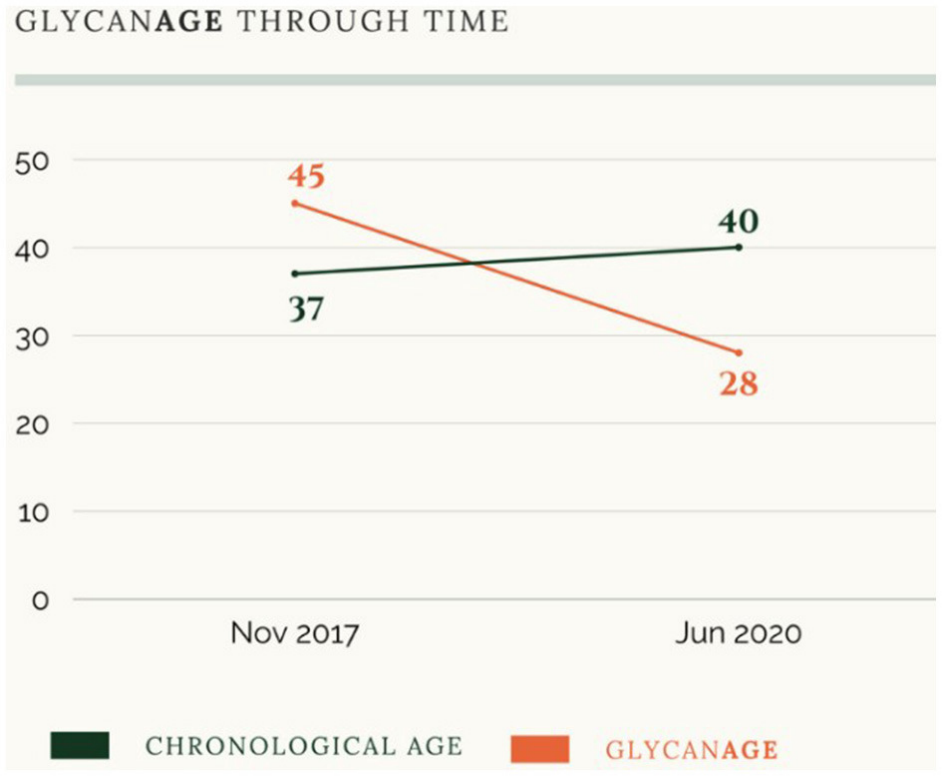
Screenshot from the GlycanAge website illustrating a person's biological age over time in comparison to their chronological age (November 2022).

The webpage also presents numerous examples of consumers who have managed to reduce their biological age through lifestyle interventions. Figure 1 illustrates a person's trajectory of decay through lifestyle intervention (red line) in comparison to their chronological age (black line) over a 2.5-year period. This figure shows how an individual was able to reduce their biological age from 45 to 28 in only 2.5 years. In other words, this person was able to slow down the speed of decay to an extent that graphically looks like one can “turn back time” and regain (molecular) youth. This indicates how a quite significant part of the aging trajectory is taken to happen through processes that are plastic and modifiable.

While healthcare technologies are promoted as means for enacting individual freedom in health promotion, the new wave of empowerment also comes with new responsibilities for citizens and questions about *who* is empowered and *how* (Chiapperino and Tengland, 2015). The biological clock designed by Genos is aimed at the aging population of the Global North, and specifically targets patients turned consumers who can afford to buy the test and take action to improve their health. Along with critical scholarship of personalized medicine (Prainsack et al., 2008; Harvey, 2009; Juengst et al., 2012; Prainsack, 2017), we argue that a consequence of the conceptualization of health maintenance at the individual level may be that those unable to enact maintenance may be deemed irresponsible and unfit to be self-governing citizens. Moreover, empowerment comes with exclusion: many people are excluded from even considering the test, either because Genos does not have the relevant data on specific populations to produce an accurate test, or because individuals do not have the financial means for testing or resources to follow health recommendations.

With this focus on lifestyle and individual behavior change, we move from the focus on molecular endo-decay to exo-decay, as the aging person becomes a responsible agent of control. What is also worth noting is that GlycanAge, as a personalized wellness technology, puts to the fore a very particular understanding of exo-decay. For Genos staff, only certain things count as “exo,” or the environment, in this form of decay (Pinel, 2022). In their website, the environmental factors emphasized as shaping the GlycanAge are individual lifestyle behaviors, such as diet or exercise, or personal life events such as divorce, stress or “the loss of a loved one”. Here again, structural and socio-economic factors, which may also shape exo-decay are invisible. By defining “exo” as individual lifestyle, Genos leaves unaddressed what Marmot (2005) termed the “causes of the causes”—that is, the socio-economic determinants that make certain lifestyles harder to achieve for some, or environmental determinants related to these such as exposure to pollution (Valles, 2018).

When the biological clock becomes a wellness technology on the market, decay shifts from being an inevitable trajectory to a plastic state that can be acted upon by empowered individuals who—through the test—gain knowledge of their personal decay and possible intervention options (Blasimme, 2021). In this scenario, the aging person is understood as responsible and seen as an agent of cause and control, capable of affecting their own malleable trajectory of aging. This particular understanding of aging echoes recent trends in aging policies, which emphasize the plasticity of aging and human agency in impacting one's aging, with for example global initiatives such as the healthy aging program developed by the World Health Organization (2015). The notion of healthy aging specifically underlines that while the rate of deterioration differs among individuals, it is modifiable through individual actions, and programs for healthy aging usually point to a set of concrete practices individuals can enact to modify their aging trajectory (Lassen and Moreira, 2014; Lamb, 2017).

In summary, while the test places individuals on an aging trajectory that inevitably ends with death, it suggests that there is significant room for interventions to slow down decay or even “turn back time” to reach a younger biological age than before they took the test. Thus, individuals can move along this trajectory if they enact change in their lifestyle. While aging becomes a malleable process, the temporality drawn here is that of an indeterminate future which is open to interventions, the actors of change being individuals acting on their lifestyle. Specifically, intervening upon the aging trajectory implies maintenance work, a responsibility which is shifted onto the shoulders of individuals. And as we show next, a particular form of maintenance work is advocated to control the forces of decay identified through biological clocks.

## Maintenance work to slow down decay

As the GlycanAge promotional materials promise, buying the test means embarking on a “wellness journey.” This starts with taking the test and being delivered results about one's biological age. Next comes a consultation to discuss the results and receive advice in order to “optimiz[e] your health and longevity.” Or as one can read on the GlycanAge website:

Our plans come with complimentary guidance for your health and wellness journey. Once you receive your results, you will be able to book your free one-to-one video consultation with a Care Team Specialist, who will work with you to discover where you may like help in improving. (GlycanAge website, November 2022)

Members of the GlycanAge Care team include a health coach, what they call a “Lifestyle Medicine doctor,” nutritionists, personal trainers and fitness instructors. In the consultation, care specialists and Genos customers discuss and interpret the results of the biological age test, but this interpretation is highly dependent on the individual providing “profile details,” or as Katja explains:

We collect also different information about your lifestyle, previous disease history, you know, medication, also stress level. Anything that could actually correlate with results. Because very often clients come to us and tell us “OK, now I get a number, like I'm 63 my GlycanAge is 83, why?” And then it's easier for us to try to… So we are not MDs [medical doctors] here. We don't claim that what we are selling is any kind of diagnostic test, but we have to look at the data that they provide us. They can choose whether they want to give this data or not. It's optional. But if they choose to give it to us, you can try to indicate what might be the reason for the result of their test and then they can choose whether they want to take that further. (Katja)

Based on the information provided by people taking the test, the GlycanAge care specialists offer an interpretation of the results. For example, a biological age result above the norm can be deemed to be linked to high-stress levels and poor nutrition. In this context, Genos staff do not hesitate to reach conclusions about causality—specifically, they establish a causal pathway between GlycanAge results and lifestyle. Such interpretation then leads care specialists to formulate advice about areas in the individual's life that could be “improved” in order to promote healthy aging. In other words, Genos and their care specialists encourage customers to convert the measurements of their biological age into actions. In line with Moreira et al. (2020) study of biological clocks, we thus observe a pragmatic engagement with the measurements, whereby they are translated into concrete practices to enhance one's aging prospects, regardless of the uncertainties about the test and which health domain the results should inform.

Concretely, much of the advice given to individuals focuses on diet. For example, their website displays tips concerning ways to prevent “weight-gain during menopause,” “anti-aging foods,” or again “anti-aging supplements.” The solution offered to a decaying body is thus maintenance through lifestyle. The emphasis on lifestyle as a course of action may be linked to the absence of more specific advice (Juengst et al., 2012). In effect, with Genos's focus on lifestyle and diet, we observe a particular form of care being advocated to slow-down decay: rather than a relational form of care like the one depicted by anthropologist Moser (2011) in her study of dementia in nursing homes where the aging individual is thought of in a web of relations, what is put to the fore is self-care while aging becomes a matter of individual and isolated practices. Individuals are made responsible through several steps. First, they are responsible for taking the test and for filling out information that could be used to interpret their test result. Second, after receiving lifestyle counseling, they are responsible for enacting the suggested changes to improve their health and wellness.

This form of maintenance work is in fact commodified, since GlycanAge offers a subscription model whereby individuals take several tests at regular intervals to monitor their biological decay and to assess the impact of the lifestyle interventions on the pace of decay. Or as the GlycanAge website states: “We highly recommend dual testing to track your progress toward slowing aging.” Katja explains this strategy:

Ideally we would actually use the GlycanAge test to monitor your speed of aging. To kind of have control over your healthy aging. You could do like the test once a year. Or if you want more information within your lifestyle change cycle, then you can do it every 6 months for example. And see whether this intervention works for you or not. Because the thing is, the same intervention might work for you but not for me or for her. … So what we are saying now is, this is your first read-out, you know, go out there in the world, make a change that you think might benefit you. And then come again for the next round of testing. If it works fine, continue with that, you know you can read that, but if it doesn't, change it and do something else and then come take a new test. So, what we will also try to develop now is some kind of subscription model where we can actually test. … Plus for us of course commercially it's good. Yes, but this is like, as I said, this is the closest to personalized medicine we have gotten so far. (Katja)

**Figure 2:**
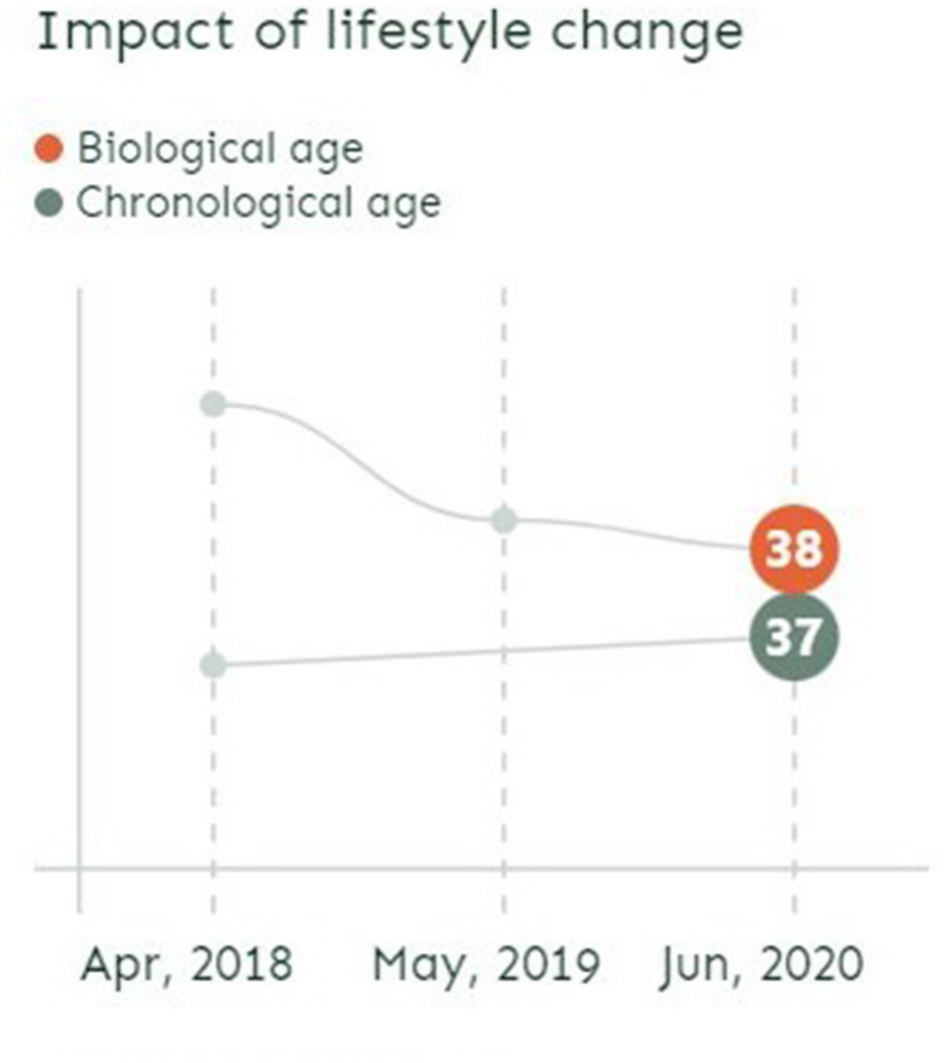
Screenshot from GlycanAge website illustrating the possibility to change biological age over time (November 2022).

The implicit claim is that taking regular tests enables individuals to keep their bodily decay in check while monitoring the impact of their maintenance work. On the GlycanAge website front page, the personal stories displayed showcase regular testing. With graphs showing two curves (one for chronological age, the other for biological age), the stories look to illustrate the significant impact of lifestyle change by pointing to important fluctuations in biological age over rather short periods of time (from half a year to 2 years) (see Figure 2).

The figures on the GlycanAge webpage present biological age as a relatively stable, but modifiable, trajectory in time. However, according to other researchers, there is another side to that story, which is that under normal conditions, biological age naturally fluctuates over time. A recent study (Komaki et al., 2022) of epigenetics biological clocks shows that epigenetic age can change by more than 3 years from day to day in apparently healthy individuals without intervention. In one customer story on the GlycanAge webpage, we also learn that a COVID-19 infection impacted the test results as he “had aged 4 years” from the initial result. This raises further questions about what is measured and about the basis for recommending and evaluating specific lifestyle interventions based on “snapshot” testing at specific time points.

In the vision articulated by Katja, the responsibility of the person taking regular tests goes even further. The individual is not just a customer following professionals' advice, but also an experimenter urged to try out different lifestyles and monitor the effect on their measured biological age. It is the responsibility of the individual to figure out what interventions work best—and buying new tests is the way to know what works. Crucially, bodily maintenance—in this vision—becomes a life-long project. Individuals are urged to keep on making adjustments to their lifestyle in order to improve their health, while they should keep taking biological age tests to check how their actions affect their aging trajectory. This particular vision of bodily maintenance corresponds to what Blasimme (2021) calls “ground-state prevention.” This form of prevention focuses on signs of declining bodily functions, rather than specific diseases, and seeks to enhance human capacities by attempting to control and postpone aging. In other words, rather than living longer or preventing specific diseases, “ground-state prevention” is about gaining healthy life-years by attacking “ground causes” of multiple age-related diseases, e.g., through geroprotective drugs or, as in our case, personalized recommendations for lifestyle changes.

From Katja's words above, we also learn how for GlycanAge staff, biological age tests come to represent personalized medicine. For them, what makes it personalized medicine is that individuals not only receive a personalized assessment of their biological decay but are also given access to specialists, who can design a tailored plan to slow down their trajectory of decay based on this information. However, the biological clock represents a very particular version of personalized medicine, as it takes the shape of a wellness technology, a commodity sold directly to individuals. In fact, the sort of personalized medicine offered by GlycanAge reminds us of what Prainsack (2022 p. 211) refers to as “boutique practice”, whereby data interpretation and personalized health strategies are available to a select few who are wealthy enough to afford the time of human experts. One is also left wondering whether GlycanAge indeed represents a more “precise” approach than traditional healthcare, where general health advice is formulated based on individual's clinical data. The GlycanAge test in itself is not suggestive of personalized interventions. Rather, the maintenance work suggested to individuals taking the test is based on staff's interpretations of biological decay, supplemented with additional information from the individual. It is also worth noting that examples of health advice featured on the GlycanAge webpage resemble general advice about healthy lifestyle. This raises questions about the necessity and basis for individualizing agency in aging *via* biological clocks.

## Discussion: The politics of preventing decay

Biological clocks are human constructions that make particular forms of decay visible, and encourage specific types of maintenance work. According to the intended purpose of the clock, researchers make decisions about what data to include, what resources to draw upon, and what epistemic approaches to aging to mobilize. When restricted to the confines of the laboratory, the biological clock brings forward a vision of aging as an inevitable trajectory of decline. However, when the biological clock moves to the market, aging becomes a malleable and plastic trajectory over which individuals can (and should) act. In other words, we come to see how biological clocks are not neutral technologies assessing decay that just “exists” within individuals' bodies, but rather are man-made technologies that bring to light particular conceptions of aging and of the aging person. As they do so, we argue, biological clocks enact a politics of decay.

In the social study of medical technologies, there has been a great interest in promise and potentiality (Brown, 2003; Martin et al., 2008). This literature directs attention to how politics of potential facilitate hope and futurity, while shaping science and financial investments (Svendsen, 2011; Taussig et al., 2013). The concept of decay helps us see that in the field of aging, such articulations of future potential are closely linked to a politics of decay. “Optimizing” or “potentializing” aging through biological clocks embody and rely on ideas about decay. In particular, the politics of decay we identify in the development and commercialization of biological clocks is concerned with the biological enclosure of existence and the dependent opposition of life and death (cf. Povinelli, 2016). While decay is an inevitable trajectory that starts at birth and ends with death, the individual endowed with life and agency has the potential to stretch time between birth and death. Biological clocks, and the maintenance work that is promoted along with them, thus articulate and bring together two distinct temporalities: one where decay constitutes an inevitable trajectory with death as the ultimate end, and one where decay can change from one day to the next and can be intervened upon, controlled and managed. Biological clocks bring these two temporalities into tension by emphasizing how individuals can stretch out time and have agency over an inevitable trajectory of decay.

Thinking about biological clocks through the lens of politics of decay helps problematize dominant norms regarding whose and what aging is deemed problematic, in what context, and who should be made responsible for managing it. It helps us see how the development of biological clocks rests on particular ways of approaching, studying and knowing decay. Specifically, our case shows how researchers bracketed knowledge about aging as either endo- or exo-processes of decay. At the Wilson Lab, researchers draw on omics technologies to build their biological clocks, and as they do so they invite the “environment” to the molecular level (Landecker, 2011). However, this is not followed up in their study and aging is bracketed as endo-processes internal to the individual. This particular way of knowing decay is unknown in the translation of biological clocks into commercial products. With GlycanAge, decay is approached as exo-processes, yet only certain things count as “exo,” specifically those related to lifestyle. Echoing anthropologist Geissler (2013, p. 16) who argues that “zones of unknowing” are created and maintained “in the pursuit of scientific knowledge,” we see how the development of biological clocks rests on particular ways of knowing decay that come together with forms of “unknowing.” What we find particularly remarkable is how the building of biological clocks in the laboratory and commercial products in the biotechnology company unknowns decay as a multifaceted and complex relationship between individual and environment. Rather, an individual's bodily decay is reduced to molecular processes which may be related to individual lifestyle, while this individual is treated as if they are cut off from their family, neighborhood or social class.

The biological clock's way of “knowing” decay turns aging into a life-long concern resulting in maintenance work over which individuals have a moral obligation and responsibility. The GlycanAge webpage presents successful examples of citizens who have benefitted from taking on this responsibility. In one of the featured customer stories, we meet Christian who took the GlycanAge test because of concerns that his lifestyle could have negative long-term impacts on his health. He was surprised to learn that his GlycanAge was seven years younger than his “actual age.”^4^ Nevertheless, he decided to implement lifestyle changes to optimize his GlycanAge and takes tests annually to monitor his progress. While the example positively shows the motivating potential of biological clocks for lifestyle change, it also illustrates how the responsibility to age “as healthily as possible” has no upper limits. The endpoint is not merely to reach the “normal” age of the baseline. Even if your molecular clock estimates that you are several years “younger” than your chronological age, there is still room for improvement. Similarly, there is no limit to the age or period at which individuals could benefit from maintenance work. Any sign of decay, detectable as invisible molecular changes in glycans, is a target for actions to optimize lifestyle. Optimization of biological age thus exemplifies how ground-state prevention is not limited to changing the aging trajectory from “pathological” decay to “normal” decay but involves an *agification of life itself* (Blasimme, 2020, 2021). Individuals are encouraged to worry about aging at an increasingly early time in life and through increasingly encompassing dimensions of measurements and age-preventive actions. Optimizing biological age is a life-long preoccupation, regardless of the admitted uncertainties about what is measured and the connection between ground-state prevention and future health outcomes.

We are left to wonder who is to benefit from such specific ways of measuring and slowing down decay. Put differently, who is the technology of the biological clock for? Decay is not always considered a problem, but rather, decay is seen as problematic at certain times, places, and when it touches certain people. On a global scale, it is in the Western world that bodily decay is seen as especially problematic and is regularly intervened upon. This can seem paradoxical since it is also in this part of the world that people live the longest and healthiest lives. It is there that geriatrics medicine has flourished over the 20^th^ century, thus turning aging into a legitimate site of medical care and prevention (Blasimme, 2021). As such, decay is deemed out of place when it touches the already healthy and wealthy of the Global North. The vision of aging enacted through the biological clocks we studied speaks to Western conceptions of aging and developed countries' approaches to gerontology that see aging as a modifiable trajectory. One can argue that technologies of biological clocks are intended to serve the already healthy individuals of developed countries, rather than improve life expectancy of the majority across the globe. This underscores how biological clocks are not neutral scientific facts emerging from the statistics of omics data but are performative technologies that shape how we view aging and health. Biological clocks are—according to those promoting and selling them—constructed through a selective focus on forms of decay which are measurable only through patented technologies, yet—it is claimed—modifiable through individual lifestyle changes. Meanwhile, efforts to make molecular signs of decay visible leave other aspects invisible, such as socio-economic aspects that affect aging and life expectancy both locally and globally.

## Data availability statement

The datasets presented in this article are not readily available because the data contain personal information. Requests to access the datasets should be directed to CP clemence@sund.ku.dk.

## Ethics statement

Ethical review and approval was not required for the study on human participants in accordance with the local legislation and institutional requirements. According to Danish legislation, study participants were orally informed about the purpose of the study and gave their consent. All data were handled and stored according to the rules of the Danish Data Protection Agency.

## Author contributions

CP led the design of the ethnographic study, collected empirical material, led the analysis and conceptualization, and led the writing and editing of the manuscript. SG and MNS acquired funding, supported the analysis and conceptualization, and supported the writing of the manuscript. All authors contributed to the article and approved the submitted version.

## Footnotes

^1^This focus differs from the metaphor of the “biological clock” in reproductive health to describe the time constraints on fertility, usually that of women.

^2^Methylation of cytosines in the cytosine-guanine nucleotide pair is the most common DNA-change in mammalian cells (Moore et al., 2013).

^3^They approach care as “persistent tinkering” to improve life by attending to people and their relations “in a world full of complex ambivalence and shifting tensions” (Mol et al., 2010: 14).

^4^ https://glycanage.com/self-care/lifestyle/ensuring-work-life-balance-and-getting-over-covid-19/
